# Brief FASD prevention intervention: physicians’ skills demonstrated in a clinical trial in Russia

**DOI:** 10.1186/1940-0640-8-1

**Published:** 2013-01-08

**Authors:** Tatiana Balachova, Barbara L Bonner, Mark Chaffin, Galina Isurina, Vladimir Shapkaitz, Larissa Tsvetkova, Elena Volkova, Irina Grandilevskaya, Larissa Skitnevskaya, Nicholas Knowlton

**Affiliations:** 1 The University of Oklahoma Health Sciences Center, 940 N.E. 13th Street, Nicholson Tower Suite 4900, Oklahoma City, OK, 73104, USA; 2 St. Petersburg State University, 6 Makarova nab, St. Petersburg, 199053, Russia; 3 St. Petersburg State Pediatric Academy, 54, Malaya Balkanskaya Str., St. Petersburg, 192283, Russia; 4 Nizhny Novgorod State Pedagogical University, 12 UlyanovaStr., Nizhniy Novgorod, 603950, Russia; 5 NSK Statistical Solutions LLC, Choctaw, OK, 73020, USA

## Abstract

**Background:**

Alcohol consumption during pregnancy can result in a range of adverse pregnancy outcomes including Fetal Alcohol Spectrum Disorders (FASD). Risky drinking among Russian women constitutes a significant risk for alcohol-exposed pregnancies (AEP). Russian women report that obstetrics and gynecology (OB/GYN) physicians are the most important source of information about alcohol consumption during pregnancy and developing effective prevention interventions by OB/GYNs is indicated. This is the first study focused on implementation of an AEP prevention intervention at women’s clinics in Russia.

**Method:**

The paper describes the intervention protocol and addresses questions about the feasibility of a brief FASD prevention intervention delivered by OB/GYNs at women’s clinics in Russia. Brief physician intervention guidelines and two evidence-based FASD prevention interventions were utilized to design a brief dual-focused physician intervention (DFBPI) appropriate to Russian OB/GYN care. The questions answered were whether trained OB/GYN physicians could deliver DFBPI during women’s routine clinic visits, whether they maintained skills over time in clinical settings, and which specific intervention components were better maintained. Data were collected as part of a larger study aimed at evaluating effectiveness of DFBPI in reducing AEP risk in non-pregnant women. Methods of monitoring the intervention delivery included fidelity check lists (FCL) with the key components of the intervention completed by physicians and patients and live and audio taped observations of intervention sessions. Physicians (N = 23) and women (N = 372) independently completed FCL, and 78 audiotapes were coded.

**Results:**

The differences between women’s and physicians’ reports on individual items were not significant. Although the majority of physician and patient reports were consistent (N = 305), a discrepancy existed between the reports in 57 cases. Women reported more intervention components missing compared to physicians (p < 0.001). Discussing barriers was the most difficult component for physicians to implement, and OB/GYN demonstrated difficulties in discussing contraception methods.

**Conclusions:**

The results supported the feasibility of the DFBPI in Russia. OB/GYN physicians trained in the DFBPI, monitored, and supported were able to implement and maintain skills during the study. In addition to the alcohol focus, DFBPI training needs to have a sufficient component to improve physicians’ skills in discussing contraception use.

## Background

Alcohol use during pregnancy is the leading preventable cause of mental retardation and can result in Fetal Alcohol Syndrome (FAS) and a wide range of Fetal Alcohol Spectrum Disorders (FASD) [1-4]. The worldwide rate of FAS has been estimated to be 1.9 per 1,000 live births [5]. Recent studies indicate a higher FAS rate of 2 to 7 per 1,000 in the US, and FASD prevalence is estimated to be 2%-5% among elementary school children in the US and some Western European countries [6]. The rates are likely to be higher in countries with greater alcohol use and limited education about the effects of alcohol consumption during pregnancy. Although the FAS rates in Russian general populations have not been established, studies report high FAS and FASD rates in Russian orphanages [7-9] and in children adopted from Russia [10].

FAS and FASD are completely preventable by avoiding alcohol use during pregnancy [11,12]. Approximately 12% of women in the US [13] and over 20% worldwide consume alcohol during pregnancy [14]. Most women eliminate or reduce alcohol consumption on learning that they are pregnant. However, approximately half of all pregnancies are unplanned, and many women are not aware they are pregnant until four to six weeks into pregnancy and continue using alcohol at pre-pregnancy levels [15]. As a result, a significant proportion of women consume alcohol during the early stages of pregnancy prior to pregnancy identification [14,16]. Studies indicate that alcohol exposure early in pregnancy may affect fetal development even if followed by later gestational abstinence [17]. A combination of at-risk drinking with the possibility of becoming pregnant constitutes a significant risk for alcohol-exposed pregnancies (AEP), and a pre-conceptional approach to preventing FASD has been recommended [18].

In Russia, nearly all women report drinking in the year before pregnancy, and depending on the study, 20%-60% drink to some extent after pregnancy recognition, and 3%-7.4% report binge drinking during pregnancy [19,20]. In a longitudinal outcome study of 413 pregnant women in Moscow, 20.2% reported binge drinking around the time of conception, and 4.8% reported binge drinking in the most recent month of pregnancy [21]. In a sample of 648 women recruited from women’s clinics in two regions of Russia, between 32% and 54% of non-pregnant women were at risk for AEP^a^[19] in contrast to 2% of non-pregnant women in a US national sample [22]. Applying comparable risk criteria, the general population at-risk rate in Russia (32%-54%) was higher then that of the highest risk women in the US, i.e., US women at drug and alcohol treatment centers US (24%) [22]. Many Russian women eliminate or significantly curtail alcohol consumption after pregnancy recognition, but minimal reduction in use occurs during the pre-conception period, even among women who are actively attempting to become pregnant [19]. Among women who were trying to conceive, 67% reported binge drinking in the prior three months [14]. The prevalence of binge drinking among Russian women who might become or are trying to become pregnant constitutes a significant public health problem.

Brief physician intervention (BPI) has been recognized as an effective approach to reducing alcohol use and related health problems in patients at risk in primary care [23,24]. Although research provides some evidence that BPI reduces women’s AEP risk [25-32], studies are needed to ascertain the efficacy of brief interventions (BI) for women and to determine the type of AEP prevention interventions that could be the most effectively implemented in primary health care [33-35]. BPI can be effective in reducing AEP risk if it is feasible, deliverable, and correctly implemented; however, feasibility has sometimes proven challenging [36-39]. In order to have a significant public health impact, intervention models must have both efficacy and deliverability. In other words, the intervention must be amenable to implementation, at scale, within authentic service systems, with reasonable fidelity and quality, and in substantial quantity. Feasibility of AEP prevention utilizing BPI in the Russian cultural context and health care system has not been studied.

Our initial studies of AEP risk in Russia used survey and interview methods with women and physicians in order to inform development of an AEP prevention strategy. Key findings included that a) the periconceptual period appeared to be a critical risk window; b) Russian women viewed their OB/GYN physicians as having perhaps the single strongest influence on their health beliefs and behaviors; c) most women already modify their drinking after pregnancy recognition, largely due to an interest in their baby’s health; and d) most women are unaware of AEP risk prior to pregnancy identification. Based on these key findings, we adapted a BPI model (to be described in detail later) and began implementing it at OB/GYN clinics in Russia. The parent study was a two-arm, 20-site, site-randomized trial aimed at evaluating the effectiveness of an intervention to reduce the risk for AEP in non-pregnant women. OB/GYNs (“women’s clinics”) were randomly assigned to intervention or control (no intervention) condition, and study participants were recruited from both intervention (10 clinics) and control sites (10 clinics). The aim of the current paper is to describe the BPI model that was developed and deployed at the intervention clinics, and present information drawn from implementation of quality control efforts about its feasibility and deliverability in the Russian context. This is the first study focused on implementation of an AEP prevention intervention at women’s clinics in Russia. Subsequent papers will examine intervention impact on downstream client level AEP risk outcomes.

## Methods

The study was reviewed and approved by Institutional Review Boards at St. Petersburg State University (SPSU) and the University of Oklahoma Health Sciences Center (OUHSC) and was conducted with approvals from the participating clinics.

### Setting and participants

The study was conducted at public women’s clinics in two locations in Russia represented by the major urban population of St. Petersburg (SPB) and more rural population of the Nizhny Novgorod Region (NNR). A total of ten clinics, five at each location (SPB and NNR), were assigned to the intervention. The clinics varied from a small rural clinic with one OB/GYN in the NNR to a large urban clinic with over 20 OB/GYNs in SPB. Commitments from the SPB and NNR Health Administrations were received to ensure cooperation from the participating clinic directors. Organizational support was obtained from the clinic directors to participate in the study and to allow participating physicians at intervention clinics to incorporate the study intervention into routine clinic visits with the study participants. Participating physicians met the following criteria: 1) certified in obstetrics and gynecology, 2) employed at least 50% time at a clinic assigned to the intervention, 3) agreed to serve in the study, 4) participated in the intervention training, and 5) demonstrated skills in completing the intervention protocol. A total of 26 OB/GYN physicians were trained in the protocol. Two did not commit to participate in the study, and one did not meet the post-training skills criteria and was removed. A total of 23 OB/GYN physicians (8 in NNR and 15 in SPB) participated in the study as interventionists. The physicians were female with a mean age of 38 years and average of 13 years in practice. Physicians were reimbursed approximately $20 per intervention.

Patient participants were recruited for the study as consecutively enrolled non-pregnant women who were at risk for AEP between July, 2009-July, 2011. Patient inclusion criteria were: a) childbearing age women (ages 18–44 years); b) fertile; c) not currently pregnant (by self-report or a test result); d) engaging in AEP risk behaviors, i.e., specifically reporting having unprotected intercourse at least once in the last six months and drinking eight or more drinks per week on average or four or more drinks on one occasion within the past three months; e) living in the area served by one of the study clinics; f) available for follow-up for 12 months; and g) providing voluntary informed consent. A plan was made to over-recruit women with higher alcohol consumption to have at least 20% of the sample score 8 or higher on the Alcohol Use Disorders Identification Test (AUDIT) [40]. A review of women’s AUDIT scores conducted after enrolling 80% of the targeted sample at each study location indicated a significant number of study participants with high AUDIT scores at the majority of clinics (N=8). At the remaining two clinics, AUDIT was administered upon screening to recruit few heavier drinkers. A total of 374 women were recruited at the 10 intervention clinics in SPB (n=197) and the NNR (n=177); 29% of the study participants scored 8 or higher on AUDIT. Participants received a gift at completion of the baseline assessment and the first intervention session (an equivalent of $25).

### Intervention protocol

The intervention was adapted from two evidence-based FASD prevention interventions, Healthy Moms [30] and Project CHOICES [41]. Results from our previous studies in Russia guided selection and adaptation of this intervention protocol. The high prevalence of AEP risk among non-pregnant women in Russia who combine at-risk drinking with a possibility of becoming pregnant [19] dictated a need in intervention that would target women prior to pregnancy, e.g., non-pregnant women of childbearing age, and addressing both behaviors that place women at AEP risk, e.g. at-risk drinking and inconsistent family planning/contraception. Women from our prior studies indicated that advice by OB/GYN physicians or nurses would be the most trusted source of information about health behaviors and alcohol consumption during pregnancy [42]. There is a well-established Russian OB/GYN health care system with services such as prenatal care and family planning/contraception services provided at district women’s clinics free of charge. Based on our prior surveys of physicians and interviews with Russian experts, it was decided that the intervention protocol should be brief, incorporated in a routine clinic visit, and should require one to two sessions maximum as it is unlikely that some non-pregnant women would return for more than one follow-up visit. This is the first intervention protocol for AEP prevention in Russia and the first protocol for a dual-focused AEP prevention intervention designed to be deliverable by OB/GYN physicians during routine women’s clinic visits.

First, we reviewed BI guidelines to make certain that the major components of effective interventions (e.g., advice, feedback, goal setting, additional contacts for further assistance, and support [24,43]) were included in the intervention protocol. Second, we reviewed FASD prevention studies and extracted elements from two evidence-based FASD prevention interventions with sound evidence for reducing AEP risk in non-pregnant women: Healthy Moms [30] and Project CHOICES [41]. Project CHOICES is a dual-focused intervention drawn from the Motivational Interviewing (MI) [44] framework and designed to decrease the AEP risk in non-pregnant childbearing age women by either reducing drinking or improving contraception or both. However, CHOICES itself could not be directly used within our intended parameters because it requires four 45 to 60 min counseling sessions with a mental health professional/counselor and one contraception session with a family planning clinician. A key reason for selecting CHOICES as one source for adaption to the Russian context was its flexibility in targeting both prevalent among Russian women problematic behaviors (risky drinking and lack of contraception) in one intervention. Also, OB/GYN physicians assess and assist women in contraception use and are in a unique position to deliver this facet of the CHOICES approach. This is the first feasibility study testing delivery a dual-focused AEP prevention intervention by OB/GYN physicians.

We adapted the structural elements from the Healthy Moms [30] protocol to make our intervention deliverable during routine clinic visits. The Healthy Moms protocol was designed for women in the postpartum period to be deliverable in two 15-min clinic visits followed by two phone calls by OB/GYNs, outpatient nurses, or research staff. Similarly to CHOICES, Healthy Moms utilizes MI and includes a patient workbook that contains results of screening and personalized feedback about AEP risk, worksheets on drinking (and contraception in CHOICES), and drinking diary cards.

The adapted protocol, which was termed the Dual-Focused Brief Physician Intervention protocol (DFBPI), implements MI principles, focuses on both contraception and alcohol use, and is designed to be deliverable routinely by OB/GYN physicians at women’s clinics. The intervention targets childbearing age non-pregnant women who are at risk for AEP, i.e., risky drinkers who are using contraception inconsistently. The DFBPI protocol and materials were prepared in consultation with Russian project consultants, obstetricians and behavioral health experts, including Russian women. The CHOICES and Healthy Moms intervention materials (e.g. workbooks) were translated and modified in accordance with DFBPI. Materials were translated and back translated by bi-lingual behavioral health experts in order to ensure that the materials were culturally congruent, accurate, and would be correctly comprehended by Russian women. A physician training protocol was developed and pre-tested by the study research group in a small randomized educational trial using a two-arm, pre/posttest design [45]. The results of this study showed that Russian OB/GYN physicians randomized to the training condition demonstrated significantly improved skills after the training.

**Figure 1:**
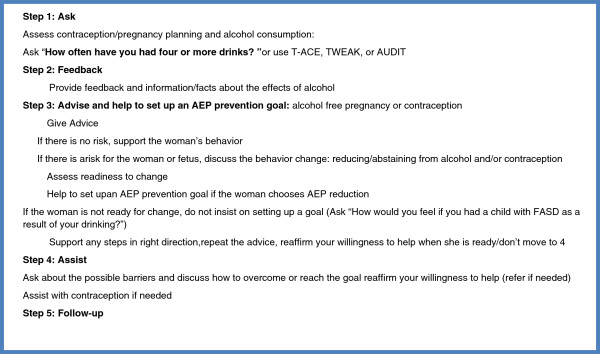
Dual-Focused BPI protocol.

The DFBPI, with the translated title, *Baby’s Health is Your Choice,* consisting of two face-to-face structured brief 5–10 min intervention sessions was incorporated in OB/GYN clinic visits scheduled approximately one month apart. Because of the lack of informational materials about contraception in Russia, an educational brochure [46] about contraception methods was also developed for the study. The DFBPI physician algorithm or steps to be taken by physicians are included in Figure 1. 

**Figure 2:**
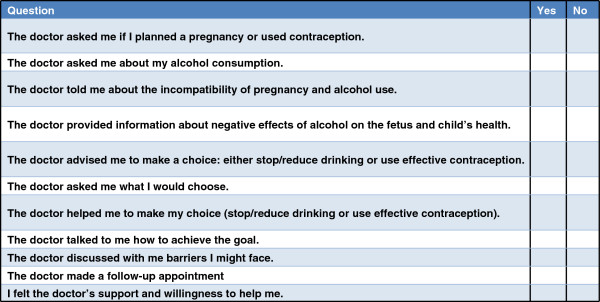
Fidelity Check List - 1st clinic visit (Women’s Form).

In the intervention condition during the first visit, a woman’s contraception practices and alcohol use were assessed and feedback about AEP risk was provided. The woman received informational materials about the effects of alcohol on a fetus and FASD, risky levels of alcohol use, and contraception methods; was provided an opportunity to discuss her options and possible barriers; was assisted in setting up her AEP reduction goal (if she chose AEP reduction); received a workbook with exercises; and was scheduled for a follow-up visit. (Key structural elements of the first intervention session protocol are included in Figure 2).

**Figure 3:**
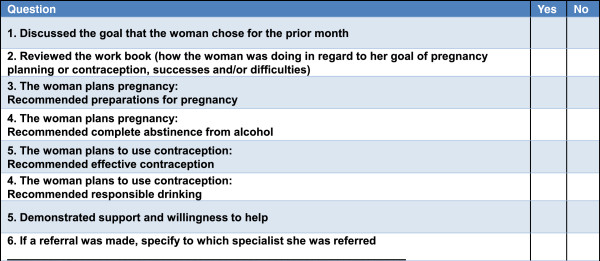
Fidelity Check List - 2nd clinic visit (Physicians’ Form).

The workbook consisted of condensed intervention messages that included 1) self-determination/feedback about the woman’s risk for AEP; 2) defining safe alcohol use during pregnancy, if attempting to become pregnant or if at-risk of unintended pregnancy; 3) recommended drinking limits if using contraception; 4) family planning/appropriate contraception use; 5) how to reduce AEP risk; 6) worksheets for self-evaluation of importance, confidence, and readiness to use alcohol safely; 7) plans for pregnancy or contraception, 8) decisional balance regarding alcohol and contraception use; 9) goal setting, and 10) a diary to record intercourse, contraception use, and alcohol use during the subsequent four weeks. The participant was asked to read information in the workbook and educational brochures about FASD and contraception, complete exercises and the diary between visits, and bring the book to the next clinic visit to discuss with the OB/GYN. The second session protocol is tailored to the woman’s choice of pregnancy planning or contraception. (The key structural elements of the second intervention session are included in Figure 3). The two DFBPI sessions were incorporated into routine OB/GYN clinic visits and could include taking a medical history, conducting a physical exam, and providing prescriptions or contraceptives if indicated. The estimated total time required for physicians to deliver the DFBPI was 5–10 min per session.

### Training and monitoring of physicians

Physicians were trained in the intervention by the study supervisors who were PhD level psychologists and a senior MD/PhD OB/GYN physician. The training included a three-hour FASD education module on the effects of alcohol consumption during pregnancy, FASD, prevention, and screening and brief interventions followed by a four-hour instructional and practice workshop. The physicians learned the DFBPI protocol, application of basic MI principles, and practiced conducting the intervention in a nonjudgmental and empathic manner. The intervention protocol was presented in a step-by-step format with instructions, and skill training techniques such as scenarios, video demonstration, and role plays, which were employed to train physicians to the behavioral competency criteria by demonstrating their delivery of all components of the 5–10 min DFBPI protocol. Feasibility and deliverability data for the DFBPI protocol were drawn from project quality control efforts. Physicians delivering DFBPI were monitored in four ways. Monitoring included reviews of the intervention fidelity check lists (see Measures section) completed by physicians and by patients after each intervention session, direct observations of physician’s intervention interactions with patients conducted by the study supervisors (at least one session was observed for each physician at the beginning of the study), and reviews of audio recordings of clinic intervention visits. The OB/GYN intervention fidelity plan required completing 80% of the components of the protocol with 90% of patient contacts. The project supervising faculty, which included PhD psychologists, an OB/GYN, and a substance abuse physician, were available to provide feedback, consult about cases, problem-solve, and provide coaching as necessary.

## Measures

The intervention fidelity check lists (FCL) were developed for this study and included the key structural aspects of the intervention protocol. FCL were completed by women and physicians independently after each clinic intervention visit. Patient exit interviews regarding clinic visits have been used in research to monitor intervention delivery and determine feasibility of interventions [47-49]. FCL were reviewed by Russian project faculty and consultants and pilot tested prior to implementation. Women’s FCL were administered in person to patients by the study research assistants immediately after the session. Examples of women’s and physicians’ FCL are included in Figure 2 (women’s FCL for the first session) and Figure 3 (physicians’ FCL for the second session). As specified by the intervention protocol, the first visit intervention components were uniform for all women while the second intervention visit components varied depending on the goals selected by the woman. The first session FCL completed by physicians and women were utilized in this implementation study.

As an additional measure to ensure that physicians maintain intervention skills over the time, audio recording of first intervention sessions was implemented in year 2 of the study. The audiotapes were coded using the FCL by two research investigators independently (85% agreement between coders). The physician’s intervention style/implementation of MI skills, including how non-confrontational/non-judgmental the physicians’ style of interaction was, how great an opportunity the patient had to set up her own goal, and how much the physician supported the patient’s self-confidence were coded on a scale 1 to 5.

### Data analysis

Categorical variables were summarized as proportions. Categorical contingency tables were analyzed with McNemar’s test if the data were paired and through a chi-square test if they were not. Continuous variables were summarized with means, standard deviations, and ranges. An alpha of 0.05 was considered statistically significant.

## Results and discussion

FCL were completed for all first clinic intervention visits (N=374) conducted by 23 physicians. Two patient FCLs were incomplete, which resulted in a total of 372 patient FCL (196 in SPB and 176 in NNR) and 23 physician FCLs utilized in the analysis.

**Figure 4:**
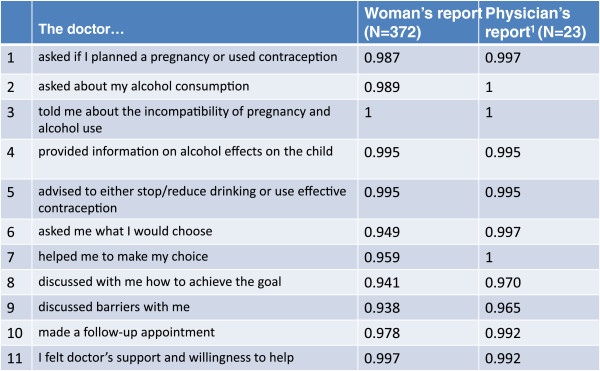
**Proportion of completed intervention components. **^1^The differences between women’s and physicians’ reports on individual items were not significant.

The proportions of completion of the intervention components by physicians’ and women’s reports are included in Figure 4.

Completion of the intervention components varied between 100% (asked about alcohol consumption, informed about incompatibility of pregnancy and alcohol use, and helped to make a choice) and 96.5% (discussed barriers) by physicians’ reports and between 100% (informed about incompatibility of pregnancy and alcohol use) and 93.8% (discussed barriers) by women’s reports (Figure 4). The differences between women’s and physicians’ reports on individual items were not significant. Physician and patient FCL reports were consistent in the majority of cases (N=307); however, some discrepancy existed between the reports in 57 cases (Table 1). Women reported more intervention components missing compared to physicians’ self-reports (p < 0.0001).

**Table 1:** **Summary of physician and patient report discrepancies**^**1**^

		**Physician (N=23)**	
		**NO**	**YES**
Patient (N=372)	NO	12	47
	YES	10	295

^1^ Discrepancies between physician and women responses were summed across all FCL questions. For example, if a patient reported Yes for every FCL question and a physician reported Yes for every question, there was agreement, which was coded as Yes/Yes (295 cases). If a patient reported Yes for every question but her physician reported No for one or more questions, that was Yes/No (10 cases). Discrepencies between physicians and patients reports were significant (p < 0.0001).

Similar results were received for a subset of interventions (N=78) by 12 physicians that were audio recoded. Out of 90 first intervention sessions completed from October 5, 2010 to July 7, 2011, 81 were audio recorded (5 patients did not consent to audio recording and 4 sessions were not recorded because of technical problems). It was not possible to code three tapes because of sound quality, resulting in a sample of 78 coded audiotapes (87% of all sessions). In 62 cases, there was an agreement between physicians’ and women’s FCL reports on whether intervention components were completed; however, in 13 cases a discrepancy existed between physicians’ and women’s FCL reports, which indicated a significant difference between physician and women’s self-reports about completed intervention components (p < 0.001). Audiotape coding indicated that physicians implemented basic MI skills, which included nonconfrontational/nonjudgmental style of interaction (94.7% out of an ideal 100% score), provided the patient with an opportunity to set up her own goals (90.7% out of 100%), and supported the patient’s self-confidence (88% out of 100%). Reviews of audiotapes of the intervention with patients revealed that discussing how to achieve a goal selected by a woman and discussing barriers were more likely to be omitted when the woman’s goal was contraception. If a woman chose reducing alcohol consumption and planned pregnancy, physicians were more likely to discuss ways to reduce/avoid drinking and possible barriers. When the goal was delaying pregnancy, discussions of choices of contraception and barriers to consistent contraception use were limited or omitted.

### Discussion

The overall pattern of results supports the feasibility and deliverability of a brief dual-focus AEP prevention model by OB/GYN physicians in Russia. The results are consistent with results of prior studies of alcohol reduction BPI and MI outside of Russia, namely that physicians will counsel their patients if they are provided skill training and quality control support [49,50]. Recruitment and participation agreement rates were high, and the majority of physicians who attended the training met skill criteria. Physicians trained in DFBPI and provided with support, individual feedback about their performance, coaching, and consultation during the clinical trial demonstrated high rates of delivery of all DFBPI components. They were able to implement the intervention and integrate it into routine women’s clinic visits. Based on reports from physicians, patients, and audiotapes, physicians outperformed the study intervention fidelity plan requirement that was set initially at 80% of the components of the intervention with 90% of patient contacts. Similarly to Babor et al. [49], both physicians’ and women’s reports indicated high performance in delivery of the intervention components. However, a discrepancy between women’s and physicians’ reports was significant with women more likely reporting omission of specific intervention components compared to physicians’ self-reports. Although there may be several explanations for the discrepancy, a review of audiotapes indicated that providers may have thought that a patient had already understood a point so they did not need to cover it much when in fact the patient did not. That was observed particularly when physicians discussed with patients contraception options and possible barriers to reducing alcohol use or utilizing contraception consistently.

Discussing difficulties/barriers that may prevent a woman from achieving her AEP prevention goal appeared to be the most difficult component for physicians to implement (or for women to grasp) and was more likely to be omitted than other components of DFBPI. In Russia, family planning and contraception counseling are conducted by OB/GYN physicians as a part of routine women’s health care. Therefore, the DFBPI training was focused more on intervention components related to alcohol consumption, which is not typically a part of OB/GYN services. The assumption was that if a woman selected delaying pregnancy/contraception as her AEP reduction goal, the OB/GYN would be equipped with skills to address her goal of improving contraception use. Unexpectedly, physicians were more likely to omit discussing methods of achieving goals and possible barriers when a woman chose delaying pregnancy. The physicians trained in DFBPI and MI basic principles to address alcohol consumption appeared to be comfortable delivering alcohol reduction intervention components of the intervention; however, they demonstrated difficulty in implementing basic MI principles to address inconsistent contraception, e.g., providing information about contraception methods and discussing options and possible barriers to improve consistent contraception use. OB/GYN physicians may benefit from expanding the contraception component of the training and developing skills to better address their patients’ contraception practices.

Strengths of this study include a relatively large sample size of 374 women and a combination of different methods that included physician and women’s self-reports completed shortly after sessions and live or audiotaped intervention observations were important for cross-validation of the results. Monitoring was conducted systematically and patient and provider FCL were obtained for all intervention sessions. Although audio recording was completed for a subset of interventions only, results indicated agreement between the audiotape and FCL data about completion of specific intervention components. Study limitations also should be considered. The intervention was a part of a clinical trial, and physicians and patients were provided with a level of quality control support that may be higher than what is found in routine clinical practice, which may limit generalization. The study was limited to physicians in public OB/GYN clinics and to the patients attending these clinics, so generalization to other service systems should be made cautiously. It is possible that some women with high AEP risk, such as alcohol-dependent women, do not seek OB/GYN or prenatal care, and it is not clear how well this AEP prevention model would serve these women. However, Russian government statistics indicate that 96.4% of women receive prenatal services from public women’s clinics [51], and therefore, the study sample represents the major OB/GYN service delivery system in Russia.

## Conclusions

This study supported the feasibility of incorporating DFBPI in routine women’s clinics visits in Russia. Physicians trained in DFBPI were able to implement and maintain the intervention skills. Despite some discrepancies between women and physicians’ reports regarding completed intervention components, there were far more congruencies, and OB/GYN physicians trained in the DFBPI, monitored, and supported during the study demonstrated performance that met or exceeded benchmarks. Broader implementation of these types of clinic-based, motivational dual-focus models outside of a research context may benefit from additional research that would determine the amount and type of quality control effort needed to obtain the highest cost-benefit. This study demonstrates that multisite implementation is feasible, but it does not establish an implementation strategy that is necessarily the most efficient. Research is needed to identify efficient ways to support implementation of AEP prevention interventions in clinical settings.

## Endnotes

^a^Risk for AEP among non-pregnant women was defined as at-risk alcohol consumption (four or more drinks on one occasion or eight or more drinks per week) plus the chance or intent to become pregnant [38].

## Abbreviations

AEP: Alcohol-exposed pregnancy (pregnancies); BI: Brief intervention; BPI: Brief physician intervention; DFBPI: Dual-focused brief physician intervention; FAS: Fetal Alcohol Syndrome; FASD: Fetal Alcohol Spectrum Disorders; FLC: Fidelity check list; MI: Motivational interviewing; NNR: The Nizhny Novgorod Region, Russia; OB/GYN: Obstetrics and gynecology (obstetrics and genecology physicians); SPB: St. Petersburg, Russia.

## Competing interests

The study was supported by Grant Number R01AA016234 from the National Institutes of Health/National Institute on Alcohol Abuse and Alcoholism and Fogarty International Center and its contents are solely the responsibility of the authors and do not necessarily represent the official views of the NIH. The authors have no competing interests.

## Authors’ contributions

All authors have made substantial contributions to conception, design, gathering data, analysis, and/or interpretation of data and have contributed to the intellectual content and writing of the article. Specifically, TB: is PI on the study and led development of the study design, the intervention protocol and materials, measures, conducted supervision, coding, participated in data analysis, and drafted the manuscript; BB: have significantly contributed to the study conception and coordination, design, and analyzing data; MC: provided significant contribution to the study design and conceptualization, participated in the manuscript writing; GI: conducted research supervision, contributed significantly to developing the study materials; VS: coordinated study and collaborated with clinics, supervised and consulted physicians, conducted training of physicians; LT: contributed to the study conceptualization and coordination; EV: conducted research supervision, contributes to developing the study materials, provided significant contribution to the study coordination; IG: coordinated clinics and conducting interventions, conducted training of physicians and supervision; LS: coordinated data collection and conducting interventions, participated in physicians supervision, prepared data for analysis; NK: participated in designing the study, performing statistical analysis, and preparing the manuscript. All authors read and approved the final manuscript.
